# Monitoring Symptoms of Infectious Diseases: Perspectives for Printed Wearable Sensors

**DOI:** 10.3390/mi12060620

**Published:** 2021-05-27

**Authors:** Ala’aldeen Al-Halhouli, Ahmed Albagdady, Ja’far Alawadi, Mahmoud Abu Abeeleh

**Affiliations:** 1 NanoLab/Mechatronics Engineering Department, School of Applied Technical Sciences, German Jordanian University (GJU), Amman 11180, Jordan; ahmed.albagdady@gju.edu.jo (A.A.); jafar.alawadi@gmail.com (J.A.); 2 Institute of Microtechnology, Technische Universität Braunschweig, 38124 Braunschweig, Germany; 3 Faculty of Engineering, Middle East University, Amman 11831, Jordan; 4 Department of Surgery, Faculty of Medicine, The University of Jordan, Amman 11942, Jordan; abeelehm@yahoo.com

**Keywords:** wearable sensors, infectious diseases, inkjet printing, screen printing, respiratory rate, heart rate, pulse oximeter, blood pressure

## Abstract

Infectious diseases possess a serious threat to the world’s population, economies, and healthcare systems. In this review, we cover the infectious diseases that are most likely to cause a pandemic according to the WHO (World Health Organization). The list includes COVID-19, Crimean-Congo Hemorrhagic Fever (CCHF), Ebola Virus Disease (EBOV), Marburg Virus Disease (MARV), Lassa Hemorrhagic Fever (LHF), Middle East Respiratory Syndrome (MERS), Severe Acute Respiratory Syndrome (SARS), Nipah Virus diseases (NiV), and Rift Valley fever (RVF). This review also investigates research trends in infectious diseases by analyzing published research history on each disease from 2000–2020 in PubMed. A comprehensive review of sensor printing methods including flexographic printing, gravure printing, inkjet printing, and screen printing is conducted to provide guidelines for the best method depending on the printing scale, resolution, design modification ability, and other requirements. Printed sensors for respiratory rate, heart rate, oxygen saturation, body temperature, and blood pressure are reviewed for the possibility of being used for disease symptom monitoring. Printed wearable sensors are of great potential for continuous monitoring of vital signs in patients and the quarantined as tools for epidemiological screening.

## 1. Introduction

Globalization is often viewed positively when viewed from technological and cultural standpoints; however, a hidden burden accompanies this unprecedented direct and indirect interaction among people and establishments worldwide. Economic development, changes in land-use patterns, an increase of travel traffic in humans and animals alike, microbial adaptation, as well as demographic changes, have been identified as contributing factors to disease emergence [1]. Information and disease surveillance play an important role in controlling pandemics and in decision-making at governmental and international levels as they aim to describe epidemiological burdens and identify and predict current and future scenarios for disease outbreaks [2]. In most countries, healthcare establishments are not designed to absorb a high influx of patients. According to Our World in Data [3], the average number of hospital beds per 1000 people was 2.70 in 2011, the lowest since 1975. This situation dictates a revolutionary change in traditional healthcare methods to keep up with the rapid increase in the human population, especially in third-world countries. Despite wide achievements in vital signs monitoring, wireless communication, and the internet of things (IoT), the world was not prepared with a solution at hand for wireless monitoring of vital signs for mild cases or home-quarantined people when COVID-19 came into existence, and most strategies utilized were very similar to those of early 20th century outbreaks. Such strategies had major socio-economic manifestations and affected businesses worldwide.

Wearable sensors offer a new definition of personalized healthcare and provide a method whereby big data and artificial intelligence can be utilized to achieve accurate estimations and risk analysis for a large number of people, impossible to process using traditional methods [4]. A complex relationship between certain vital signs and other health metrics can be linked after collecting a large amount of data, and wearable sensors can facilitate data collection and measurement of health parameters. Such sensors are expected to be transportable, completely standalone without stationary units, and cheaper than traditional measurement methods [5]. However, current wearable sensors are yet to be improved to medical-grade level and yet to solve critical issues such as durability, repeatability, wearability, and the ability to extract the maximum amount of clinical information with the minimal number of sensors [4]. Many devices have been designed for different parameters such as heart rate [6,7], body temperature [8,9], motion tracking [10,11], blood pressure [12,13], respiratory rate [14,15], and sweat analysis [16,17]. Fabricating sensors that are small, highly flexible and/or stretchable, and conductive faces other challenges in special equipment requirements and clean environments. Fabrication techniques such as photolithography [16], electrospinning [18], spray deposition [19], laser patterning [20], and printing [21] differ in dimensional limitations, accuracy, speed, and cost, and each has its advantages depending on the required level of accuracy, accessibility to certain equipment, or fabrication costs.

Several earlier works have demonstrated the use of sensors or thermal cameras for mass screening of patients [22,23,24]. A study by Radin et al. [25] has demonstrated their ability to collect resting heart rate and sleep data from 47,249 users, and as a result the data significantly improved ILI (influenza-like illness) predictions in all five states where data collection took place. Mohammadzadeh et al. [26] have recently reviewed 277 articles on wearable sensors for vital signs monitoring in epidemics and have concluded that wearable sensors present the greatest potential in disease control and early diagnosis. In order to summarize earlier works on infectious diseases and possible printed monitoring devices for future pandemics, we review infectious diseases that are most likely to cause a pandemic and categorize them in terms of symptoms, clinical management, and transmission. We also review flexographic printing, gravure printing, inkjet printing, and screen printing as methods for sensor fabrication, and select respiratory rate (RR), heart rate (HR), oxygen saturation (SpO_2_), body temperature (BT), and blood pressure (BP) sensors from the literature for comparison and evaluation.

## 2. Infectious Diseases

### 2.1. Overview

Infectious diseases are disorders caused by infectious organisms, often a kind of microorganism that invade the human body and spread from one person to another, or, in some cases, from animals to humans. Microorganisms capable of causing disease and impairing the natural functioning of the host are called pathogens [27]. These infectious agents are categorized into five main types: bacteria, viruses, fungi, parasites, and prions, and each infectious organism has specific properties that display its interaction with the human host [28]. The immune system responds to infection by producing specific antibodies targeting specific antigens to eliminate alien pathogens [29].

### 2.2. Epidemics

Epidemic diseases of the 21st century are a potentially serious threat due to the ease and quick spread of infectious agents and the enormous number of affected people. Prevalence of infections goes through three stages, beginning with an outbreak when infections increase in a relatively limited area, then infection escalates to an epidemic as a result of the sharp increase in covered areas and populations. Eventually, the epidemic can be transmitted to cover multiple territories, and this will be defined as a pandemic [30]. Emerging infections can be defined as newly recognized infections or those that have existed previously but see a sharp increase in incidences amongst a human population, caused by pathogens that have selective advantages to infect new human hosts [31]. In contrast, ‘re-emerging infections’ are infections that have previously spread and declined but now are reappearing resulting in an increase in incidences or the extension of an infected geographic zone [32]. Several aspects contribute to the emergence and re-emergence of infectious diseases, categorized into genetic, biological, social, political, and economic factors [33]. Unprecedented travel frequency, starting with the emergence of commercial airlines, had a significant role in spreading diseases such as acute hemorrhagic conjunctivitis in 1981 and SARS in 2003 [33,34]. Currently, the priority diseases updated by the WHO that pose an enormous threat to the public health with epidemic potential include COVID-19, Crimean-Congo hemorrhagic fever, Ebola virus disease, Marburg virus disease, Lassa fever, Middle East Respiratory Syndrome (MERS), and Severe Acute Respiratory Syndrome (SARS), Nipah and henipaviral diseases, Rift Valley fever, Zika and “Disease X” [35]. In this review, diseases associated with respiratory and cardiovascular symptoms are discussed because of the capability of applying various wearable printed sensors for vital signs monitoring. 

#### 2.2.1. COVID-19

In December 2019, the world witnessed the emergence of a new respiratory tract infection in Wuhan, China caused by a novel Coronavirus [36,37]. The new Coronavirus disease (COVID-19) is primarily transmitted through respiratory droplets [38,39]. When a patient who is showing symptoms of coughing or sneezing is in proximity, surrounding people are at risk of infection through exposed mucosa or conjunctiva [40]. Furthermore, the infection may also be transmitted through fomites in the infected person’s environment [41]. Once infected, the symptoms appear after a mean incubation period of 5.2 days [42], with fever, cough, and shortness of breath being the most common clinical symptoms [43,44]. Less common symptoms may include loss of taste and smell, sputum production, headache, hemoptysis, diarrhea, dyspnea, and lymphopenia [45,46,47,48]. The primary diagnostic test for COVID-19 is either through tests that identify the virus itself, as in the real-time reverse transcriptase-polymerase chain reaction (rRT-PCR) test, or through antibody detection (serological tests) [49,50,51]. The most accurate sample for virus testing is sputum, followed by nasal swabs, both samples resulting in a positive rate ranging from 42.9% to 61.1% [52]. Although some patients develop asymptomatic infections, the disease can still be transmitted, making it challenging to track and control the spread of the disease [39,53]. Although RT-PCR tests can detect the virus at an early stage of infection, previous infections can go undetected. Currently, the WHO does not recommend antibody rapid diagnostics tests but encourages the establishment of their use in disease surveillance and epidemiological research [54]. Many companies and research groups have successfully developed a vaccine, such as the Pfizer/BioNTech and Moderna [55]. Antipyretic therapy is used for fever treatment, expectorants for non-productive cough, and oxygen therapy is used with patients who have developed a severe acute respiratory infection, distress, hypoxemia, or shock [56,57]. Clinical management of COVID-19 requires monitoring of respiratory rate (RR), heart rate (HR), and oxygen saturation. In previous studies [58,59], HR and RR have been used to detect severe respiratory illnesses. Oxygen saturation is considered an important physiological parameter for COVID-19 patients which reflects the status of their respiratory and circulation systems. Clinical studies have concluded that the ratio of patients with RR > 24 breaths per min (bpm) was significantly different between ICU care and non-ICU care patients with COVID-19 [60].

#### 2.2.2. Crimean-Congo Hemorrhagic Fever (CCHF)

A new disease was discovered in the 1940s that causes a severe form of hemorrhagic fever with a fatality rate of up to 40% [61,62]. The virus responsible for the disease is the Crimean–Congo hemorrhagic fever virus (CCHFV), which is a negative-sense RNA virus that belongs to the family Nairoviridae within the Bunyavirales order of viruses [63]. The principal vector of this disease are ticks from the Hyalomma genus which are mainly found in Africa, the Middle East, and South-East Europe [64]. CCHF transmission to animals is through infected tick bites, while animal-to-human transmission is through exposure to infected livestock especially in farmers, slaughterhouse workers, and veterinarians, as well as by tick bites [62]. In contrast, human-to-human transmission is introduced through direct contact with infected patients’ blood or body fluids. Improper sterilization of medical equipment and reuse of injected needles have also been reported in hospitals causing further spreading of CCHF [65]. Symptoms start to develop after an incubation period of 1-3 days (12 days maximum) if a tick bite was cause of infection or 5-6 (maximum 13 days) in case of contacting infected blood or tissue [66]. These symptoms usually include fever, muscle pain, dizziness, neck pain, backache, headache, sore eyes, and photophobia. nausea, vomiting, diarrhea, abdominal pain, a sore throat, rash, mood swings, and confusion [62]. Moreover, hepatosplenomegaly and multi-organ failure are reported [67]. CCHF diagnosis can be achieved by virus isolation using cell culture, IgG and IgM antibodies detection using enzyme-linked immunosorbent assay (ELISA), Serum neutralization, antigen detection, and quantitative PCR using RT-PCR assays [64,68] At present, no licensed vaccine against CCHV is available. However, blood transfusion, crystalloid, and fresh frozen plasma should be provided to patients as a supportive therapy. Furthermore, Ribavirin therapy and immunoglobulin therapy are widely used and reported in the literature, but their efficiency has been controversial [69,70]. High body temperature is found in the majority of fatal cases and should be monitored regularly [71]. In addition, cardiovascular changes may occur, including tachycardia (fast heart rate), bradycardia (slow heart rate), and low blood pressure [62,72].

#### 2.2.3. Ebola Virus Disease (EBOV) and Marburg Virus Disease (MARV) 

Ebola and Marburg viruses belong to the family Filoviridae, enveloped within a single-standard negative-polarity RNA virus [73]. Human-to-human transmission is through direct contact with blood and other bodily fluids of infected persons or contaminated objects, especially in the severe stage of acute illness when high viral load is expected [74]. These viruses can be transmitted to humans through direct contact with living or dead infected animals such as bushmeat (e.g., fruit bats, chimpanzees, gorillas, forest antelopes) [75,76]. Fortunately, the Ebola virus is not airborne and is unlikely to be transmitted through respiratory drops under normal circumstances. However, from a close distance, bigger droplets from highly infected individuals could transmit the virus [77]. Despite the high fatality rate of 24–90%, EBOV and MARV are not contagious before symptoms onset [75,76,78]. The incubation period ranges from 2 to 21 days for the EBOV and from 3 to 21 days for the MARV [79,80] Symptoms are initiated with high fever, sore throat, muscle pain, fatigue, and headache. This is followed by gastrointestinal symptoms such as diarrhea, vomiting, bleeding complications, and abdominal pain. At later phases, patients develop fluid loss and hemorrhagic manifestations, whereas patients with the MARV disease also develop nervous system problems [80,81]. Clinically, it may be hard to diagnose EBOV and MARV via symptoms as these are shared with other diseases such as malaria, typhoid, and meningitis. The most common diagnostic methods include ELISA, antigen-capture detection, serum neutralization test, RT-PCR, electron microscopy, and virus isolation by cell culture [76,78]. No vaccines are currently available for either disease, but there is an FDA license for a prevention vaccine named rVSV-vectored for EBOV disease [82]. Initially, broad-spectrum antibiotics, anti-emetic or opiate medication, non-steroidal anti-inflammation drugs can be used. Rehydration with oral or parenteral fluids, oxygen therapy, renal replacement therapy, and immunomodulators have been employed as potential treatment. Furthermore, anti-coagulants have been used to prevent hemorrhages [78,80,83]. Body temperature, heart rate, respiratory rate, blood pressure, peripheral oxygen saturation, level of consciousness, body weight, and point of care glucose are recognized by the WHO as essential signs for assessing the risk of developing complications [84,85].

#### 2.2.4. Lassa Hemorrhagic Fever (LHF)

Lassa virus belongs to the family Arenaviridae and is enveloped with a segmented negative-sense RNA virus [68]. Mastomys nataliensis (multimammate rodent) is the natural host for the virus. Although infected rats do not show signs of illness, they transmit the disease to humans through their fluids, blood, and feces. Besides, human-to-human transmission occurs via direct contact with infected persons, or their blood and other bodily secretions, in addition to contaminated food ingestion [86]. Furthermore, sexual relations, infected mothers, and pregnant women present significant risk factors, while airborne transmission among humans is not reported [86,87]. The incubation period of the disease ranges from 2 to 21 days [87]. The symptoms of the initial stage are high fever and general weakness associated with malaise. The second stage’s symptoms include sore throat, headache, muscle and chest pain, vomiting and nausea, diarrhea, abdominal pain, productive cough, conjunctivitis, proteinuria, anemia, and low blood pressure. At the last stage, facial swelling, convulsions, bleeding complications, and disorientation occur [88]. IgM or IgG antibody or viral protein detection by ELISA, nucleic acids by RT-PCR and virus isolation by cell culture are the most common methods today for diagnosing Lassa virus [86,89]. At present, no licensed vaccines are available. However, early supportive care is meaningful, which is associated with oxygen therapy and rehydration with appropriate fluids. For supportive treatment, Ribavirin antiviral drugs have been used which have immunomodulatory and broad-spectrum antiviral effects [87,90]. LASV prevention is categorized into rodent control and individual prevention. Rodent control works by reducing human contact with rodents using efficient methods to keep them away from and around homes, such as maintaining good hygiene and keeping cats [86]. Body temperature, fluid and electrolyte balance, blood pressure, heart rate, and respiratory rate, in addition to other laboratory tests such as Hemoglobin and WBC (White Blood Cells) count, should be regularly carried out for illness progression status [91,92].

#### 2.2.5. Middle East Respiratory Syndrome (MERS) and Severe Acute Respiratory Syndrome (SARS)

Middle East Respiratory Syndrome Coronavirus (MERS-CoV) and Severe Acute Respiratory Syndrome Coronavirus (SARS-CoV) are the pathogens responsible for SARS and MERS, which belong to the beta-CoVs and are enveloped with a novel single-stranded, positive-sense RNA genome [93,94,95,96]. Dromedary camels are the main host for MERS-CoV, although it may have originated in bats [97]. Camel to human transmission occurs through exposure to infected camels either by direct contact or less directly by exposure to their mucus droplet, aerosol, feces, urine, drinking troughs, ingesting unpasteurized milk or raw meat, and drinking urine. The Chinese horseshoe bat is the natural reservoir for SARS-CoV which transmits to the human via exposure to infected animals and their food. Human-to-human transmission for MERS-CoV and SARS-CoV occur directly via airborne, droplets transmission and contacting eyes, nose, or mouth, and less directly by fomite transmission. However, MERS-CoV human-to-human transmission is not sustained, but mutating to become more efficient in transmission is also possible [94,98,99,100,101]. There is no evidence for infected mother to newborn transmission by intrauterine route [102,103], but stillbirth has been reported for pregnant women with MERS-CoV [104]. Similar to COVID-19, the incubation period of MERS-CoV and SARS-CoV is up to 14 days [105]. The symptoms of MERS and SARS range from asymptomatic or mild infection to severe acute pneumonia with acute respiratory distress syndrome. Both diseases may begin with fever, chills, rigors, dry or productive cough, myalgia, shortness of breath, sore throat, and headache. Further, gastrointestinal symptoms including diarrhea, abdominal pain, vomiting and nausea, and anorexia may be seen. Severe infections may also occur, including acute respiratory distress, multi-organ failure, and shock, especially among old patients. Leucopenia, lymphopenia, and thrombocytopenia are also reported [93,99,101,106,107,108]. The standard method for diagnosis of MERS-CoV and SARS-CoV is viral nucleic acid detection by RT-PCR assay for respiratory tract samples. Antibody detection for a serum sample by positive screening assays (ELISA, indirect fluorescent antibody IFA) is also used for diagnosis but should be followed by a neutralization assay for confirmation. Antigen detection assays can also be used, but are not recommended according to WHO guidelines [94,106,109,110,111]. Unlike COVID-19, both SARS and MERS have more serious fatality rates of 34.4% and 11% for MERS and SARS, respectively [112,113]; fortunately, their spread was not as wide as COVID-19 and the number of confirmed cases for SARS and MERS is much lower than that for COVID-19. At present, no licensed treatments are available for MERS-CoV and SARS-CoV [97,114]. However, early supportive therapy plays an important role which includes oxygen therapy, continuous treatment with intravenous fluids, and the presentation of empiric antimicrobials. Ribavirin, Interferons, and lopinavir/ritonavir show potential as antiviral regimens [96,110]. In terms of clinical management, body temperature, oxygen saturation, respiratory rate, blood pressure, pulse rate, bodily fluids and sepsis should be monitored, in addition to other laboratory tests [110,115,116,117].

#### 2.2.6. Rift Valley Fever (RVF)

Rift Valley Fever Virus (RVF) belongs to the family Bunyaviridae that is enveloped with a negative-sense single-stranded RNA genome [118,119]. Mosquito genera like Culex and Aedes are the main transmitters of the RVF [120]. Mosquito to either human or animal transmission, especially to domestic ruminants, occurs via bites from infected mosquitos. Animal to either human or other animal transmissions also occurs through direct contact with infected animals and their bodily fluids, blood, other secretion, or fomites, in addition to ingesting unpasteurized milk from infected animals. Nevertheless, human to human transmission has not been reported, thus outbreaks in urban areas unpredictable. The incubation period ranges from 2 to 6 days [120,121,122]. Vertical transmission between pregnant woman and newborn is possible [123]. Most patients with RVF show no symptoms, or a form of mild illness including fever, weakness, headache, dizziness, and pain in muscles, back, and joints, with, in some cases, vomiting, abdominal pain, and a sensitivity to light; however, in these cases the diagnosis might be mistaken for meningitis. Limited cases develop more severe illnesses including neurological disorders, vision loss, and hemorrhagic fever syndromes with bleeding manifestations [120,122,123,124,125]. The case fatality rate (CFR) for the disease is below 1% for most cases, however, in patients who develop hemorrhagic fever this can rise to 50% [120]. The virus diagnosis can be achieved by virus isolation in cell cultures, specific detection of IgG and IgM antibodies using ELISA, and viral nucleic acid detection by real-time RT-PCR assays which are recommended due to their sensitivity and reproducibility [122,126]. Currently, no licensed vaccine is available and no specific treatment for RVF patients should be used, as most cases are mild [120]. Blood, fresh frozen plasma, and albumin should be transfused intravenously for patients in the initial phase, as necessary. Moreover, antibiotics are administrated to patients against bacterial superinfection [120,125,127,128]. Body temperature and blood pressure are crucial clinical features to be continuously monitored [123,129].

#### 2.2.7. Nipah Virus Disease (NiV)

Nipah virus (NiV) belongs to the family Paramyxoviridae within the genus Henipavirus viruses and is a single-stranded negative-polarity non-segmented RNA genome [130]. The case fatality rate for this disease is significantly high at 40–75% depending on clinical management efforts and other factors [131]. The fruit bat is the natural host of the NiV. Animal to human transmission occurs via direct contact with infected pigs, horses, or fruit bats and their secretions, in addition to ingesting raw date palm sap and uncovered water that could have been contaminated by a fruit bat. On the other hand. human to human transmission can occur through direct contact with patients’ respiratory secretions and urine or via aerosols due to close contact [131]. The incubation period of NiV ranges from 3 to 14 days or more in some cases [130,132,133,134]. The symptoms vary from sub-clinical to mild to severe, or sometimes even fatal. At the initial phase of NiV infection, patients present mild to high fever accompanied by headache and muscle pain. Later, patients develop dizziness, drowsiness, and altered mental status associated with unconsciousness and disorientation and may progress to seizures and coma in severe cases [131]. Patients at the initial phase may also develop acute respiratory distress syndrome along with cough, vomiting, diarrhea, and sore throat [135,136,137]. NiV diagnosis can be achieved by viral nucleic acid detection using RT-PCR, and IgG and IgM antibodies detection using ELISA [138]. At present, no licensed vaccine is available for NiV and patients are subjected to supportive care [130,139,140]. The signs of fever and respiratory infection require continuous measurement of body temperature, oxygen saturation, and respiratory rate [141].

Table 1 differentiates between the covered infectious diseases in terms of pathogens responsible for the infection, CFR, modes of transmission, symptoms, diagnosis, vaccine, and clinical monitoring.

### 2.3. Infectious Diseases in the Literature

**Figure 1:**
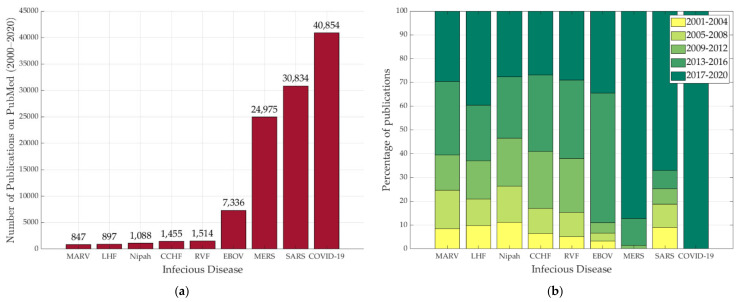
Statistics on publications for each disease for the period of 2000–2020, (Source: PubMed search results; search terms: “COVID–19”, “severe acute respiratory syndrome”, “middle east respiratory syndrome”, “ebola virus disease”, “rift valley fever”, “Crimean-Congo hemorrhagic fever”, “nipah virus”, “lassa fever”, “marburg virus disease”). (**a**) Number of publications mentioning each disease from the year 2000 to 2020; COVID-19 shows an unprecedented spike. (**b**) Percentage of published research on each disease after normalization during five four-year periods to show the periods at which certain topics received the highest interest.

The number of publications on infectious diseases has seen a rapid increase over the past two decades; however, COVID-19 produced an unprecedented number of scientific publications in less than a year. COVID-19 reached almost every country in the world and affected the most robust economies, which encouraged governmental, non-governmental establishments and companies to invest and provide millions in funding to scientists to develop vaccinations, testing kits, and patient health surveillance systems. Figure 1a (data and MATLAB code are available in the Supplementary Materials) shows the number of publications from 2000–2020 on the aforementioned infectious diseases according to PubMed’s search results. By far, COVID-19 achieved the highest research interest of all infectious diseases. Being respiratory infectious diseases, SARS and MERS have also gained a huge interest following the COVID-19 pandemic, as approximately 70% of publications mentioning MERS were published in 2020. The Ebola outbreak in 2013–2016 that was responsible for nearly 28,600 cases [145] has also provoked interest corresponding to the epidemic period, as can be seen in Figure 1b. Other infectious diseases including RVF, CCHF, Nipah, LHF, and MARV have a significantly low number of publications considering that the WHO classifies them as priority diseases for R&D. In general, all diseases in this study did not seem to attract sufficient research interest before outbreaks occur; as a result, the world was not ready when COVID-19 came to existence. Research funding providers should dedicate special awards for preparing and fighting epidemics in the medical, engineering, social, and education aspects, even after COVID-19 is defeated. What is currently occurring, from huge funding capabilities to high research interest in COVID-19, is viewed positively amongst the scientific community, as it enables scientific and social research groups to better understand the dynamics of epidemics while an enormous amount of data can be used to build models or predict outcomes.

### 2.4. Disease Surveilance

**Figure 2:**
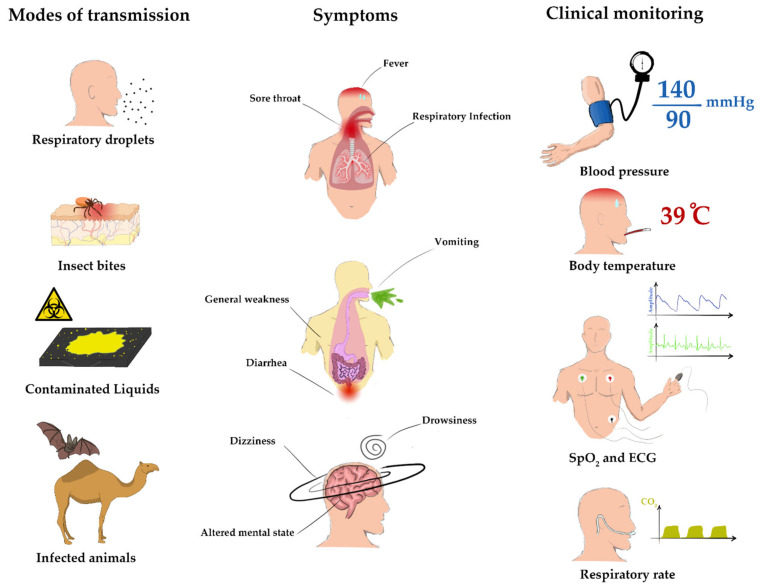
Summary of modes of transmission, symptoms, and clinical management for the infectious diseases mentioned in this study. Symptoms are categorized into three categories: respiratory, digestive, and nervous.

Common symptoms can be found between diseases, some of which can be directly measured by investigating vital signs such as measuring body temperature to detect a fever and measuring respiratory rate to detect respiratory infections; however, not enough data is currently available to examine how vital signs development can help in diagnostics or in accumulating a better understanding of disease development [146]. A study by Churpek et al. [147] showed the value of vital signs trends in improving the accuracy of detecting critical illnesses, as vital signs trends are often overlooked in disease detection models. In this matter, it is essential to discuss the possibility of recording vital signs for each patient in hospitals on a timely basis and whether hospitals should invest in cloud storage services, servers, and data loggers to generate big data for further studies and diagnostics tools. Data shows that an overlap of symptoms can occur between multiple diseases [148], making it challenging to differentiate between diseases or diagnose judging only on symptoms without necessary laboratory tests; however, different diseases may exhibit certain patterns in disease progression or symptom development that require a large amount of data to be well-articulated. Hospital vital signs monitors can measure multiple parameters such as blood pressure (BP), body temperature (BT), oxygen saturation (SpO_2_), heart rate (HR), respiratory rate (RR), end-tidal CO_2_ (ETCO_2_) and electrocardiogram (ECG). The frequency at which vital signs are assessed depends on severity [149]. Modern hospitals use reliable and accurate bedside monitors with EMR (Electronic Medical Records) integration capability to enable digital processing of data and patient documentation; however, the high implementation costs, need for computer and technical skills, and workflow changes limit the widespread use of EMR software in low-income countries [150]. Figure 2 gives a summary of the main modes of transmission, symptoms, and clinical management of the aforementioned infectious diseases. Recent advances in wireless wearable sensors has attracted huge interest in recent years, enabling commercialized solutions for convenient wireless health tracking [151]. Smartwatches have become popular and often include a heart rate sensor, a skin temperature sensor, and a pedometer for health tracking and activity tracking. These measured parameters can be used for diseases surveillance and prediction as demonstrated by Mishra et al. [152] for COVID-19 pre-symptomatic detection. However, smartwatches are usually used for fitness purposes rather than clinical, which restricts their use. Although smartwatches can provide reliable data for some cases, specialized wearable physical and chemical sensors can measure other parameters such as blood glucose, sweat PH and circulating metabolites and nutrients in sweat [153,154]. The miniaturization and low fabrication costs of these standalone sensors can facilitate the collection of big data from continuously recorded vital signs, shifting the burden on hospitals to acquire similar technologies as they lean more towards personalization. These sensors and their different fabrication techniques will be discussed in the following section.

## 3. Wearable Sensors

During the past two decades, the field of wearable and flexible sensors has witnessed accelerated attention due to the continuous need for durable, accurate, and low-priced sensors to be worn by people daily without triggering discomfort or inconvenience, especially for real-time disease monitoring [155]. A major contributor to this momentum is the advancement in fabrication techniques allowing a wide variety of methods with different resolutions and costs. 

### 3.1. Printing Technologies

Various techniques can be used to deposit a conductive ink onto specific parts of a substrate, therefore defining the sensor area. These techniques can be divided into contact and non-contact methods. Contact methods involve direct contact between the substrate and the printing medium which has a physical design to describe the features to be printed, as in flexographic or screen printing [156]. On the other hand, non-contact methods usually use a digitized design to directly control an actuation mechanism in order to deposit ink in predefined locations without touch with the substrate, as in inkjet or aerosol jet printing [157,158]. Each method has advantages. Design can be changed very easily in non-contact methods, as it usually relies on a digital file such as an image or vector file, but in contact printing a physical mask must be fabricated for each design, which can be costly and time-consuming. However, the throughput of non-contact printing is not comparable to contact printing which can be more suited to the industrial needs of the mass fabrication of hundreds of thousands of sensors [159]. Lithography, flexography, gravure, and screen printing represent conventional printing technology which has a common working principle, in which a contact pressure must be applied to transfer the ink in the contact zone between the printing plate and the substrate as a layer of ink in two-dimensional patterns, with residual ink remaining on the printing plate [160]. 

#### 3.1.1. Flexographic Printing

Flexography is a printing method via which ink is transferred to a substrate through a process that begins with a fountain roller that continuously feeds low-viscosity ink from the ink path to an anilox roller that is evenly screened with cells to hold a specific amount of ink controlled by a doctor blade to sweep the excess [161]. Furthermore, a consistent thickness of ink is evenly transferred to the printing cylinder that attaches to a rubber or photopolymer plate. By a slight contact pressure of the impression cylinder, the ink will be transferred from the printing cylinder to the substrate. Along with various substrates such as poly(ethylene terephthalate) (PET) and SU8-coated substrates used for printed electronics (PE) applications, a wide range of conductive inks can be printed, such as Ag and CNTs. However, applications with high pattern resolution and micro-scale size have limitations due to the high-speed roll to roll, de-wetting process, and rely on continuous uniform lines rather than on merging disconnected dots, which leads to defects in the conductive lines, making an open or overlapped line, in addition to waviness. Thus, controlling the pressure load and cavity aspects ratio is important to achieve proper waviness and avoid destruction of the underlying layers [162,163]. Combinations of micro-contact and flexographic printing show potential to realize fine solid lines in a micro-size equal to 10 µm using PDMS (Polydimethylsiloxane) as a printing plate [164]. 

#### 3.1.2. Gravure Printing

Gravure printing is an ideal method for mass printing that relies on a steel-based cylinder (also known as gravure cylinder) electroplated with copper to reduce the wear from the ink transfer and contact with the substrate [165]. The gravure cylinder contains the design features as engraved cavities, which are achieved using laser engraving or chemical etching. The quality of gravure printing is highly dependent on the ink’s surface tension, appropriate pressure between the gravure printing roller and the impression roller, printing speed, and the wettability of the substrate and the ink. The thickness of the printed layer is defined by the volume of the cells on the gravure cylinder [159,166]. The typical resolution of gravure printing is in the range of 70–250 µm [167]. Enabling large-scale and cost-effective printed electronics for wearable sensors and health monitoring devices, the use of gravure printing to print on flexible substrate using Ag nanoparticle-based ink has been reported in several cases [168,169]. On the other hand, gravure printing on stretchable substrates using a plate-to-roll gravure method and the effect of different process parameters have also been discussed in previous studies [170,171,172].

#### 3.1.3. Screen Printing

Owing to cost-effectivity, simplicity, compatibility with a wide range of functional inks and substrates, versatile two-dimensional pattern design, scalability, and robustness, screen printing is the most promising conventional mass-printing technology for stretchable and flexible devices [173,174]. Screen printing started at the beginning of the 20th century and mainly consists of an image carrier and a squeegee. The principle of the printing process is the forcing of a relatively high-viscosity ink through a screen with porous mesh, often made of plastic, natural silk, or metal fabric. The image is defined on the mesh by a photochemically developed stencil; therefore, the ink is imprinted on the substrate through the mask created by the image. According to the ink’s solvent, the material of the mesh and stencil are determined [162,175]. The ink is swept relative to the screen using the edges of the squeegee, where the front edge forces the mesh to bend in order to contact the printing substrate and to make sure that the ink is adhering to the substrate. Screen printing inherently allows the application of a very thick layer of ink that is defined by the thickness of the stencil. The printed line width can be in the range of 30–100 µm [160,176]. Thus, when high conductivity electronics are needed, screen printing has been demonstrated as an appropriate technology. Two techniques are commonly used for screen printing, namely flat-bed screen printing and rotary screen printing with an essential difference in the working principle. The former has a flat printing plate that consists of a mesh held by a screen frame and a flat substrate that is securely held during the printing by the base plate. This technique has assorted advantages such as an inexpensive printing plate, the capability to adjust when the substrate is integrated with other electronics, compatibility with making one print at a time, in addition to printing on a very large area on the scale of 10 m^2^. On the other hand, the rotary printing method uses a cylindrical printing mesh where the ink is contained inside the cylindrical printing plate, and will be imprinted on a continuously fed substrate using a fixed internal squeegee and an impression cylinder. The advantages of the rotary technique are summarized as high printing speed and throughput, achievable wet thickness, and attractive edge resolution [160,176,177,178]. The quality of screen printing for conductive lines typically depends on three parameters. Firstly, the screen/mesh properties i.e., fineness of the mesh, stencil thickness concerning the clearance between the mesh and the substrate, and the degree of the opening which is the percentage of all opening mesh in proportion to the entire mesh surface. Secondly, we must consider the ink and substrate properties i.e., rheological properties of the printed ink in which the ideal viscosity behavior is high rest viscosity, low viscosity at high shear, and fast viscosity recovery time. As for the substrate, decreasing the substrate surface energy leads to a decrease in the wettability of the printing ink, which improves the printed line resolution. The third parameter is the squeegee’s features, i.e., shape, speed, and pressure [177,178,179,180,181]. However, in terms of printing resolution, screen printing has limited resolution in contrast to gravure and flexographic printing; nevertheless, screen printing is the most straightforward in terms of complexity [182]. Assorted types of ink such as silver nanowires (AgNWs), carbon nanotubes (CNTs) and such graphene-based ink, can be used for the fabrication of screen-printed electronics due to direct pattering on substrates as PDMS, thermoplastic polyurethane (TPU), PET, Tattoo, letter, nano-fiber papers, and other plastic sheets [183]. 

#### 3.1.4. Inkjet Printing

Inkjet printing is a promising non-contact technology that has been widely used in the context of wearable printed electronics for health monitoring with a printing resolution down to 20 µm [162,184,185].several attractive features, such as digitally arranging the location of ink droplets on two-dimensional substrates, compact and real-time adjustment systems as a result if having the printing images as digital data allowing modifications in diverse software, versatile patterns and multilayers printed with high resolution, and compatibility with a wide range of substrates owing to the non-contact and low processing temperature, allowing printing on a non-planer and rough substrates. Moreover, conservation of materials, cost-effectiveness, simplicity, accuracy, modularity, and commercial availability have also been identified as advantages of inkjet printing [186,187,188,189,190]. The working principle is based on the ejection of a specific quantity of low viscosity ink inside a chamber from a nozzle system through a kind of actuator that transfers to the chamber, making a contraction, in which the ink droplets are squirted at precise locations onto the substrate [186,191]. Inkjet printing techniques can be classified into continuous inkjet printing (CIJ) and drop-on-demand inkjet printing (DoD) [189]. According to the nature of the ink’s flow from the nozzle, the CIJ generates a continuous stream of liquid that is broken regularly into unique uniform droplets with equivalent spacing as a result of piezoelectric actuator vibration, which is charged based on the image. This technique can be used when high-speed printing is required, in which a 80–100 µm droplet diameter can be obtained [188,192]. However, CIJ is not a common technique for manufacturing and research purposes due to material wastage and inevitable contamination because of ink recycling [193]. In contrast, the DoD technique can be used to eject single drops from the nozzle as small as 9 µm [194]. The generation of the ink droplets can be achieved using a thermal actuation or piezoelectric actuation [195,196]. The thermal actuation relies on making bubbles inside the chamber using a thin film heater to increase the temperature of the ink above its boiling point. As a result of this, bubbles collapse creating a rapid expansion and pressure pulse to generate the ink droplets through the nozzle [191,195,197,198]. On the other hand, piezoelectric actuation relies on applying a sudden and quasi-adiabatic contraction to the chamber wall by a piezoelectric transducer that controls the frequencies for ejecting ink droplets through the nozzle [199]. To achieve high-resolution conductive patterns using inkjet printing, two strategies are discussed extensively in a previous work [200]. The first strategy increases the footprint resolution resulting in minimizing the distance between the printed dots and lines. The second modulates the solute behavior of the ink by modifying its constitution, substrate, and other properties to obtain a different depositing distribution. Moreover, the crucial effect of selecting the proper solvent and substrate are investigated in [201]. Various flexible and stretchable substrates can be used for the fabrication of conductive patterns, that include elastic polymers substrate such as polydimethylsiloxane (PDMS) and polyurethane (PU), paper-based substrate such as temporary tattoo paper (TTP), thermoplastic polyurethane (TPU), organic flexible paper, and polymeric substrates such as polyethylene terephthalate (PET), polyimide (PI), polyethylene naphtholate (PEN), and polycarbonate (PC) [187,202,203,204,205]. The inks and their fluidic properties represent a significant function for achieving optimal inkjet printing performance. Thus, various inks have been reviewed recently, such as nanoparticle inks, organometallic inks, conductive polymers, graphene oxide, and carbon nanotubes [206,207,208,209]. However, understanding the fluidic characteristics of the ink droplets, optimizing the features of the inkjet printer elements such as the nozzle, realizing the materials’ chemical and physical properties, and embedding multi-technologies and electronics are represented as essential challenges for inkjet printing technology [189,210].

**Figure 3:**
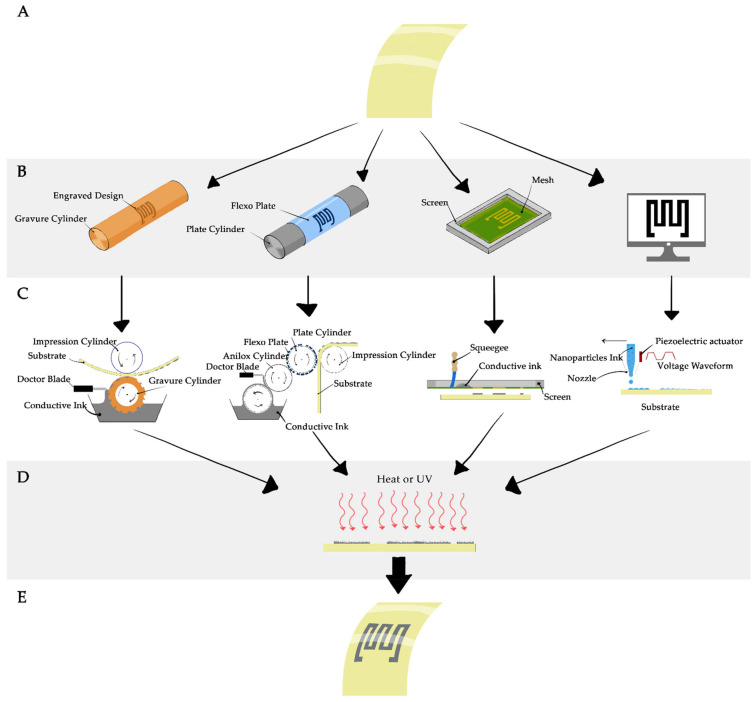
A summary of the printing methods described above (gravure, flexographic, screen, and inkjet printing). Step (**A**) represents the substrate preparation, step (**B**) shows the feature realization needed to transfer the design from the carrier, step (**C**) illustrates the process of different printing methods, step (**D**) shows the sintering process were solvents evaporate and the sold components of the ink remain, and Step (**E**) shows the finished printed sensor. Steps (**A**,**D**,**E**) are usually common in all printing techniques.

Regardless of the fabrication technique, each printing method goes through five main steps. Figure 3 summarizes the aforementioned printing techniques as steps from A-E. Step A describes the substate preparation that includes choosing the best substate to ensure good adhesion between the substrate surface and the chosen ink. This step may include hydrophilization of hydrophobic substrates like PDMS through plasma or chemical etching. Step B illustrates the design and feature realization, but in inkjet printing this step does not include preparing a hardware mask or a carrier, while other methods may require a process of fabrication or machining. Step C illustrates the actual printing process while step D shows the sintering process at which the printed pattern is exposed to UV light, or heated to a certain temperature until the solvent is evaporated. Finally, in Step E we reach the final representation of the printed features.

## 4. Vital Signs Monitoring for Infectious Disease Symptoms 

### 4.1. Body Temperature 

Human body temperature is an important indicator that reflects the body’s health and provides crucial information as the body raises its temperature as a response to an infection or inflammation due to various diseases [211]. As noted in the infectious diseases section, fever is the common symptom for the proposed infectious diseases, defined as a regulated rise in human body temperature above the normal range (36.5–37.5 °C) to reach an optimal level for host defense [212,213,214,215]. However, there is a possible rational conclusion that fever may be protective for patients with infection [216]. A rise in body temperature is not always a fever but could be an indication of heat production increase while heat dissipation decreases at the same time, and this is based on duration which may be classified into acute (less than 7 days), sub-acute (up to 2 weeks), and chronic (more than 2 weeks). Human body temperature can be categorized into Core Temperature (T_c_), which is the temperature of the internal organs, and Shell Temperature (T_s_) which is the skin temperature. T_s_ may be influenced by external circumstances within the range (31.5–35.3 °C). In addition, the common sites for non-invasive core temperature measurements in clinical settings are the sublingual site, axilla, and tympanic membrane in the ear [213,217]. Considering the portability and ease of use of non-contact temperature measurement devices and forehead scanners, their reliability is in debate as their accuracy depends on environmental conditions, physiological variability, hormonal changes, scares, or body fat [218,219]. Fever is a significant symptom of infection, thus, a community-wide campaign was implemented in Taiwan for body temperature monitoring, in order to improve early detection of possible SARS as the first sign [220,221]. Consequently, continuous monitoring and follow-ups of fever and febrile response are essential in the understanding of several diseases and infections. Several research groups and companies have developed wearable body temperature sensors [222]. However, sensors that utilize a printed sensing film have an advantage when it comes to conformality to the human body and flexibility. Therefore, printed wearable temperature sensors present an attractive solution due to their real-time, continuous, and remote monitoring via a comfortable attachment to the skin surface, in addition to cost-effective temperature mapping. In [223], a fully printed temperature sensor was developed on PEN substrate with a high-performance sensing layer of PEDOT:PSS with crosslinker of (3-glycidyloxypropyl) trimethoxy silane (GOPS) and a non-ionic surfactant Triton TX-100, with an encapsulation layer of fluorinated polymer passivation (CYTOP), to enhance the humidity stability and temperature sensitivity of the developed sensor. The sensor was tested using a temperature and humidity chamber to demonstrate a high sensitivity of −0.77% °C^−1^ in temperature ranging from 25 °C to 50 °C, and stability in environmental humidity ranging from 30% RH to 80% RH. Inkjet printing and dispensing printing technologies were used to develop an Ag electrode based on AgNP and the proposed sensing layer, respectively. Besides, screen printing was used along with surface mounted technology (SMT) to fabricate an integrated flexible hybrid circuit with a Bluetooth module for wireless temperature monitoring. When the sensor attaches to a volunteer’s arm, the resistance of the sensing film decreased immediately with temperature and then became stabile at skin temperature. Eventually, a mobile application was developed as a platform to receive, display, and store the detection temperature results on an android smartphone. Another promising work by Ali et al. [224] reported a simple yet effective screen-printed carbon body temperature sensor utilizing the sensitivity of carbon’s resistance towards human body temperature. Interdigitated electrode fingers of silver nanoparticles were inkjet-printed for optimum sensitivity and linearity of the sensor in the range 20–50 °C. At these temperatures, the resistance change of the silver electrode is negligible relative to the change in the carbon’s resistance, given an optimal design.

To compare previous works on body temperature measurement, a standardized definition of sensitivity must be agreed upon for all previous studies. For resistive sensors, the sensitivity in a linear region would be:

(1) $$Sensitivity=\frac{R_{2}-R_{1}}{R_{1}\;\left(\Delta T\right)}$$

where $R_{2},\;R_{1}$ are the resistance values at the temperature difference ΔT. Wang et al. [223], Ali et al. [224], Dankoco et al. [225], and Yamamoto et al. [226] have calculated the sensitivity according to this equation. However, Han et al. [227] estimated the sensitivity in A/D (Analog-to-digital) conversion values when achieving a sensitivity of −31/°C without listing the A/D resolution, therefore their sensitivity value is not be compared with other works. 

Body temperature measurement on flexible or stretchable substrates presents a significant challenge as any bend can be interpreted as a change in temperature if not properly designed. This feature in other applications may be favorable as will be thoroughly discussed in the respiratory rate measurement section. Hence, addressing this issue plays a major role in determining the maximum bending radius at which accurate temperature readings can be attained with acceptable drift. Wang et al. [223] proved to have the highest working radius of 2 mm, where only 1% of resistance change was observed. 

Table 2 lists selected previous works on printed temperature sensors. It is clear from this that using silver on its own as a sensing element produces low sensitivity, Carbon, on the other hand, produces a mid-range sensitivity, while PEDOT:PSS results in a very good sensitivity as Wang et al. [223] demonstrated. A unique work by Yamamoto et al. [226,228] scored the highest sensitivity by utilizing both carbon nanotubes and PEDOT:PSS as active elements. ECG (electrocardiogram) measurement was also included in a compact form attachable to the skin. Capacitive sensors also possess a significant advantage over resistive temperature sensors as there could be a configuration where no galvanic connection is needed for remote measurement, as Voutilainen et al. demonstrated [229]. In that work, temperature is measured by a combination polymer-based capacitor and coil made from silver paste forming an *LC* circuit where resonance frequency is measured as a function of temperature, enabling low power wireless measurements. Wang et al. [223] achieved wireless monitoring by screen printing the conditioning circuit with a Bluetooth Low Energy (BLE) module powered by a sheet battery. 

**Figure 4:**
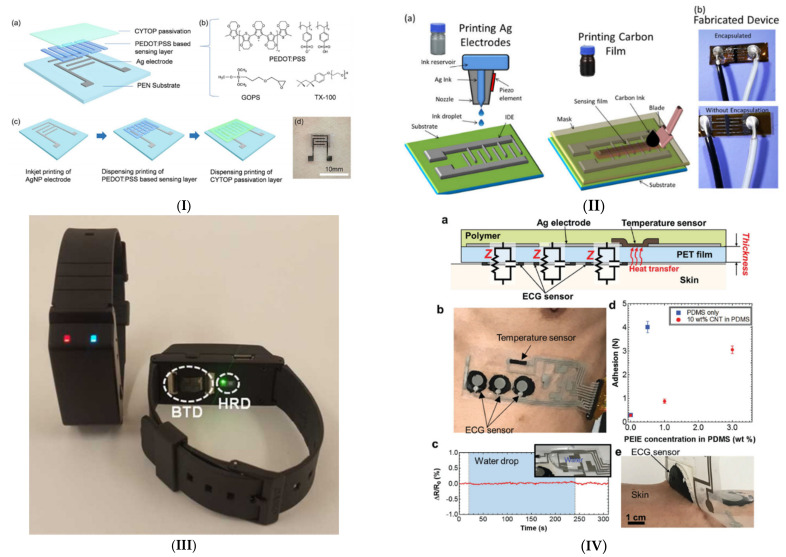
Selected printed wearable temperature sensors. (**I**) Inkjet-printed PEDOT:PSS sensor by Wang et al. [223] used under the terms of Creative Commons Attribution 4.0 International license, (**II**) Wrist carbon-based temperature sensor by Ali et al. [224] used under the terms of Creative Commons Attribution 4.0 International license, (**III**) Resistive PEDOT:PSS sensor fixed on a watch-style band by Han et al. [227] used under the terms of Creative Commons Attribution Non-Commercial License, and (**IV**) Resistive chest CNT sensor by Yamamoto et al. Reused with permission [226,228] Copyright (2017), John Wiley and Sons.

The attachment position and mechanism are essential especially in temperature sensing, as perfect skin contact must be established to achieve repeatable and accurate measurements. Eshkeiti et al. [230], Wang et al. [223] and Yamamoto et al. [226,228] managed to directly adhere the printed substrate onto the skin, resulting in more comfortable and convenient wear, while Han et al. [227] placed the sensor on a wrist band, similar to smart health monitors. Figure 4 shows notable temperature sensors with potential to be used for continuous fever monitoring.

### 4.2. Respiratory Rate

Respiratory rate is an important vital sign that represents an indication of potential cardiorespiratory diseases, such as pneumonia, cardiac arrest, and congenital respiratory disorders [231,232,233].This is important for detecting sleep disorders and monitoring infants for Sudden Infant Death Syndrome (SIDS) [234]. Relative changes in respiratory rate assume a better role in differentiating between patients in either stable or risk condition owing to a noticeable change in the respiratory rate rather than a change in blood pressure and heart rate [235]. The normal range of the respiratory rate is age-dependent. For the preterm and newborn age groups, normal respiratory rate is in the range of 40–60 and 30–50 bpm, respectively, while for the healthy children in the first two years it is recorded in the range of 20–40 bpm [236,237,238]. According to Flenady et al. [239], non-respiratory compromised healthy adults have a normal respiratory rate in the range of 12–20 bpm, while the mean value for the elderly is about 19 bpm. Therefore, a decline in respiratory rate is shown from birth to early adolescence in contrast to an increase in respiratory rate in older adults [238,240,241,242].

Abnormal respiratory rate is an important indicator of infection and other serious illnesses, as mentioned later. Thus, adult patients with a respiratory rate of more than 24 bpm should receive close and frequent monitoring, and patients showing a high respiratory rate of more than 27 bpm should receive immediate medical care [243]. Respiratory rate monitoring can be achieved using two methods: contact and non-contact respiration monitoring [231,244]. Examples of contact methods include respiratory sound using flow measurements, respiratory airflow using acoustic measurements [245], air temperature measurements [246], air humidity using relative humidity measurements [247], chest and abdomen movements using strain measurement [184], or Capnography by measuring the concentrations of CO_2_ in the respiratory gases [248]. Respiration rate can also be indirectly measured by electrocardiogram (ECG) and photo-plethysmogram (PPG) signal [249]. The contact monitoring method depends on a direct contact sensing element that is attached to the subject’s body to a certain parameter; however, the main disadvantage of this method is the need to attach the sensing element to the subject’s body, and the facial area in some cases. In contrast, non-contact respiration monitoring depends on a contactless sensing element such as radar or imaging techniques, making it more comfortable for the patient and more accurate in measurement [250]. However, the main concerns of this method are the high cost and the significant influence of the subject’s movements. Hence, there is an urgent need for comfortable, portable, low-weight, user-friendly, accurate, remote, and cost-efficient sensors for respiratory rate monitoring in continuous mode.

Given the importance of respiratory rate measurement, it is often overlooked by clinical staff in hospitals as the least commonly recorded vital sign amongst the four others, despite being considered a crucial parameter in the development of Early Warning Systems (EWS) [243,251]. Problems with equipment and measurement devices have been pointed out as one of the main reasons for the poor documentation of respiratory rate continuous monitoring [252,253]. Due to the availability of flexible, stretchable, and biocompatible substrates such as skin-friendly attachments to the patient’s body, and recent advancements in sensor fabrication techniques, printed flexible and stretchable sensors represent a promising solution for continuous respiratory rate monitoring that is accurate, sensitive, durable and cost-effective [254]. 

In earlier works [184,255,256], Al-Halhouli et al. for the first time developed and clinically evaluated a wearable inkjet-printed respiration rate sensor, made from PDMS to ensure high stretchability, biocompatibility and conformity to the human body. The sensor acts as a strain gauge sensor by utilizing the resistance increase of printed silver nanoparticle patterns when subjected to strain. A fabric belt was chosen as the attachment mechanism whereby the sensor is fixed to the chest area and records the chest movement at inhalation and exhalation, resulting in an analog signal whose fundamental frequency represents the respiration rate. The gauge factor was calculated as an indicator of sensitivity according to this equation:

(2) $$GF=\frac{(R_{2}-R_{1})/R_{1}}{S}$$

where $R_{1}$ is the resistance before applying the strain and $R_{2}$ is the final resistance, while S is the applied strain. The sensor was clinically evaluated on human subjects in various postures and fixture positions, and more recently was tested on COPD patients [257]. However, the main limitations are the small number of test subjects and sensitivity to motion.

A variety of specialized sensors have been developed to be fixed on facemasks [258], usually relying on changes in temperature and humidity between inhaled and exhaled air, as facemasks form a microenvironment where temperature and humidity are primarily influenced by respiration. Yang et al. [259] developed a screen printed nanofiber-based pressure sensor that can measure both heart rate when fixed on the chest and respiratory rate when fixed to a facemask. Screen printed AgNW (silver nanowire) electrodes on PVDFNM substrates of poly(vinylidene fluoride) and a porous TPUNM (thermoplastic polyurethane NM) dielectric layer in between work as a parallel-plate capacitor whose capacitance changes according to the applied force from breathing air. The gaps in the dielectric layer, its hydrophobicity, and the hydrophobicity of the PVDF make the sensor air permeable, and it does not accumulate moisture from breathing after prolonged usage. Cao et al. [260] used a similar approach in developing a screen-printed self-powered nanofiber-based triboelectric sensor (SNTS) by utilizing the triboelectric effect. The developed sensor has the great advantage of being self-powered since electrical energy is harvested from the applied pressure. The sensor, fabricated as an arch structure, consists of two layers: a 40 µm friction layer (PVDFNW) and an opposite friction layer (PVDFNW and AgNPs). These two layers have a difference in tribo-polarities, thus, the contact between them will charge the surface of the PVDFNW layer, printed with AgNPs, by an equal amount of positive and negative triboelectric charges. Furthermore, the AgNP layer is grounded through a load, so the electrons move to balance out the difference between the PVDFNM and AGNP layers. Su et al. [261] have also developed a facemask respiratory rate sensor, but instead of measuring the pressure induced by breathing, humidity sensing was adopted using a gold nanoparticle (GNP)-based sensor. The fabrication process for this sensor begins by printing an interdigitated pattern of carbon electrodes using a robotic dispensing system. Next, a thin film of GNPs was deposited using inkjet printing technology over the interdigitated electrodes as a sensing material for the humidity. As a result, the resistance of the sensing material decreases exponentially when the humidity increases. Lu et al. [262] took a different approach and fitted a temperature/humidity sensor inside a breathing tube, whereby the test subject is requested to breathe through the tube to record changes in humidity and temperature. The sensor consists of an interdigitated printed layer of silver nanoparticles coated with a functional layer of graphene nanoparticles as a semiconductive material with high thermal and electrical conductivity. The sensor is then connected to a commercial impedance analyzer to measure the impedance, while the Cole-Cole impedance model was used to verify the impedance characteristics, and the dielectric constant and the conductivity is changed and monitored during breathing. In another work presented as a conference paper by Mohapatra el at. [263], researchers fixed a moisture sensor under the nostrils, decreasing the resistance of interdigitated electrodes. Table 3 shows a summary of these multiple printed respiratory rate sensors.

**Figure 5:**
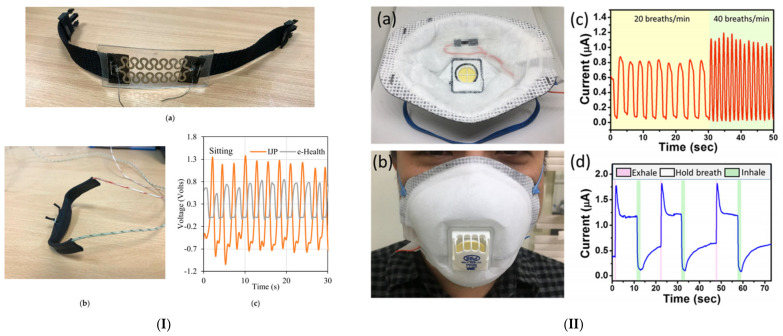
Selected printed respiratory rate sensors. (**I**) the inkjet-printed strain gauge sensor, along with a reference airflow sensor for results validation [184,255,256], used under the terms of Creative Common CC BY license. (**II**) Gold nanoparticles humidity sensor embedded in a facemask, reused with permission [261]. Copyright (2019) American Chemical Society.

Each design and detection mechanism has its advantages and disadvantages. The strain gauge belt-shaped sensor [184,255,256] is more convenient and therefore more likely to be worn by people for prolonged periods, but movement artifacts would disturb the sensor readings without a proper signal processing algorithm to rule out the undesired noise. Facemask sensors are not affected by movement, but when discussing wearability, convenience becomes a key factor. People generally do not prefer wearing masks unless they are strictly obligated to do so, let alone using a facemask for home quarantined patients to measure their respiratory rate, where they would be alone and with no other need to wear a facemask. Research materials on continuous wearable respiratory rate measurement are not as diverse as for 0other vital signs, and so developing such solutions is highly needed, especially as the world is witnessing the prevalence of respiratory infectious diseases such as COVID-19. Printing technologies can indeed cut down the fabrication costs of such sensors compared to traditional technologies like lithography. Figure 5 shows some promising printed respiratory rate sensors from the literature.

### 4.3. Heart Rate

Heart rate is a major health risk indicator for cardiovascular and pulmonary diseases, such as hypertension, hyperglycemia, tachycardia and chronic obstructive pulmonary disease (COPD) [264,265]. A study by Karjalainen et al. [266] showed that during a fever, heart rate increased by 8.5 beats per minute for every 1 °C increase of body temperature. Normal heart rate is within the range of 60–100 beats per minute (bpm). Usually, lower heart rates indicate an efficient muscular performance, which is why athletes can have heart rates as low as 30 bpm [267]. Normal heart rate also differs by age, gender, lifestyle, physical activity, and other parameters [238,268]. Therefore, it is physiologically important to be acknowledged on the patient’s history and personal information before evaluating heart rate abnormalities. Recent studies of COVID-19 patients reported that 56% of hospitalized patients with fever have developed bradycardia (low heart rate) [269]. At first glance, their results seem to contradict earlier works reporting that an increase in heart rate follows an increase in body temperature [266]; however, researchers in [269] used the term “relative bradycardia” to describe a drop in heart rate after taking into account the increase expected from fever. On the other hand, other infectious diseases such as Ebola virus disease cause mild tachycardia (increase in heart rate), where patients recorded 93±14 bpm (mean ± SD) upon admittance [269]. Hospitals measure heart rate by electrocardiography (ECG), where the electrical activity of the heart is measured directly from electrodes fixed on the skin at specific locations. An ECG signal consists of P, Q, R, S, and T waves, which contain information about heart functionality. For example, the PR interval represents the time needed for an electrical signal to transit from the sinus node contracting the upper heart chambers (atria) through the atrioventricular (AV) node contracting the lower heart chambers (ventricles), therefore reflecting the AV node’s performance. First-degree heart block can be defined at PRc (heart rate corrected PR interval) > 200ms [270]. Hospitals usually adopt 12-lead ECG devices as the golden standard, but even though 12-lead devices provide more information, the differences between 3-lead and 12-lead devices proved clinically irrelevant upon investigation [271].

The oxygen saturation level is also considered a health indicator as it represents the amount of oxygen-saturated hemoglobin in blood as a percentage. Therefore, it can reflect the quality of the respiratory system and allows symptoms like shortness of breath to be quantifiably measured. The normal range is around 95%–100% and a value below 94% is considered as hypoxemia [272,273]. There are two main methods of measuring oxygen saturation level. The first is the arterial-blood gas test, invasively drawing a blood sample from the radial artery, then testing it in blood-gas analyzers. This method returns what is known as arterial oxygen saturation (SaO_2_) as well as other parameters. The second method is a non-invasive approach called pulse oximetry where two light-emitting diodes (LEDs) at 660 nm and 940 nm wavelengths and a photodiode are placed opposite to each other across a tissue, usually on a finger; then. the light absorbance is measured for each different wavelength to eventually produce a pulsating signal that follows the heartbeat. Oxygenated hemoglobin absorbs more light than its deoxygenated counterpart under infrared (940 nm), resulting in a lower intensity received by the photodiode, while deoxygenated hemoglobin absorbs red light, allowing infrared to pass [274,275]. This phenomenon can be used to build a cheap and simple optical sensor for estimating the percentage of oxygenated hemoglobin in the blood. The saturation level measured with this method is called the peripheral oxygen saturation SpO_2_. Although SaO_2_ is more accurate than SpO_2_, most manufacturers of pulse oximeters claim an accuracy only of ±2% [276]. A recent study showed that SpO_2_ underestimated S_a_O_2_ by more than 3% in the majority of test subjects [277]. Changes in SpO_2_ do not predict equivalent changes in SaO_2_ in a reliable manner for the critically ill. It is a powerful method, but awareness of its limitations is essential [278]. The signal from a pulse oximeter is called a photo-plethysmogram (PPG) and can also measure heart rate because the blood volume in capillaries changes according to the heartbeat which directly corresponds to the intensity detected by the photodiode.

In this study we also consider PPG sensors like heart rate sensors. Therefore, heart rate and oxygen saturation level sensors are discussed. Many research groups have developed printed ECG patches and pulse oximeters via various techniques. Most notably, a work by Yamamoto et al. [228] was mentioned in the temperature monitoring section. The authors claimed to have fabricated a gel-less screen-printed ECG sensor from silver on PET film, whereby electrodes were printed on the bottom layer and leads were printed on the top layer. However, weak contact between electrodes and skin can affect the signal. The same research group proposed a solution for the adhesin problem by mixing poly-ethylenimine(PEIE) and PDMS with carbon nanotubes(CNTs) because adding PEIE to PDMS can increase the adhesion force to the skin [226]. CNTs provided conductivity to the silver electrode on top, while the diameter of the PEIE-PDMS-CNTs mixture and its CNT concentration affected the amplitude of the signal recorded (R-wave). Eventually, a 20mm electrode with 10 wt% CNT was proved to provide a good, readable ECG output. Furthermore, the authors have claimed in the Supplementary Materials that P, Q, R, S, and T waves were observed when attaching the electrodes to a different region. More tests on a bigger population are needed to fully characterize this sensor. Vuorinen et al. [279] also developed a screen-printed ECG sensor from silver. A four-electrode configuration was implemented with hydrogel on electrodes to enhance skin connectivity. The main advantage of this work is the comprehensive study of male and female subjects in different postures when comparing the developed bandage-like sensor with commercial electrodes. One of the main challenges for ECG measurement is the effect of motion artifacts due to the motion of muscles and the soft tissue that the electrode is fixed onto. Vuorinen et al. [279] addressed this issue and performed experiments when test subjects were lying still, standing, walking, and running. Subsequently, the ECG outputs from the printed electrodes and commercial electrodes were compared; both contained a low-frequency noise at motion while the power of the noise correlated to the motion intensity; however, the noise was higher in the printed electrode. Yamamoto’s sensor may have an advantage in this respect because the electrodes adhered well to the skin, but no motion tests were performed. 

**Figure 6:**
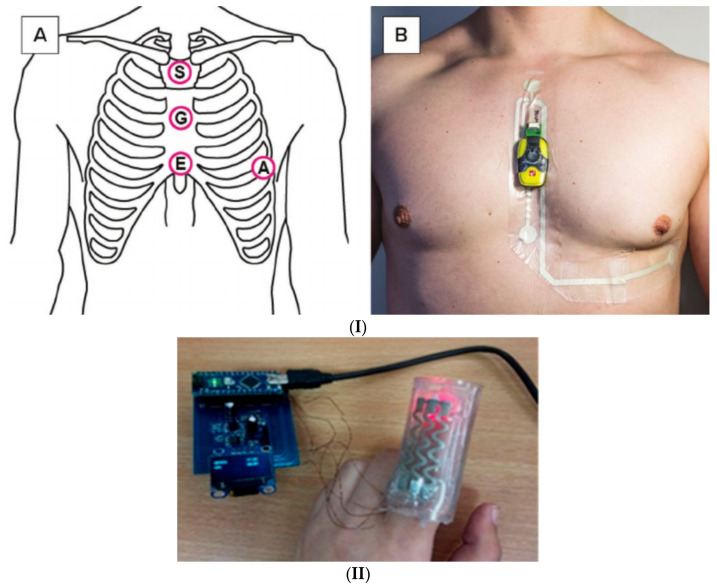
Printed heart rate and oxygen saturation sensors. (**I**) shows the screen-printed ECG sensor on TPU film; the left figure shows the placement of the 4 electrodes, while the figure on the right shows a volunteer wearing the device; reused with permission from [279], Copyright (2019), John Wiley & Sons. (**II**) shows the inkjet-printed pulse oximeter while performing live monitoring on a human subject [188], used under the terms of Creative Common CC BY license.

Abu-Khalaf et al. [188] developed a fully stretchable inkjet-printed pulse oximeter for heart rate and SpO_2_ measurement. SMD (surface-mount device) red and infrared LEDs and a photodiode were fixed on a stretchable printed silver pattern that connects to a microcontroller. Different line widths, number of cycles, amplitudes, and shapes of the printed silver pattern went through an optimization step to choose the design that ensured the best conductivity under strain. Red infrared LEDs and a photodiode were connected to the printed pattern with Galinstan liquid metal, then the entire sensor was coated with PDMS to protect the printed lines and hold the liquid metal in place. The sensor was compared with a commercial pulse oximeter and the resulting error was ±4.72% for HR and ±0.755% for SpO_2_ measurements; however, the sensor was tested only on one healthy person, and further testing on ill patients is required for accuracy, wearability, and durability. Instead of using SMD LEDs, Lochner et al. [280] fabricated organic LEDs (OLEDs) and a photodiode (OPD) from organic material through spin-coating and blade coating. The authors used red and green LEDs instead of red and infrared, because solution-processable near-infrared OLEDs suffer from lower efficiencies. This sensor is very promising, and the use of organic LEDs can lead the way to ultra-thin and robust pulse oximeters. The sensor also showed a low error of 1% for heart rate and 2% for oxygen level. Both sensors developed by Abu-Khalaf et al. [188] and Lochner et al. [280] are accurate in laboratory settings; however, both need to be validated on a wider sample with both healthy and ill subjects. Figure 6 shows some printed wearable heart rate and SpO_2_ sensors from the literature.

An ultrathin pulse oximeter was fabricated by Yakota et al. [281] with polymer LEDs (PLEDs) and an OPD, resulting in a device thickness of only 3 µm. Such a device has high potential for its integration abilities and wearability. Nonetheless, SMD LEDs are currently available only in small packages, rendering them negligible when coated or embedded in a stretchable circuit. Table 4 shows a comparison list for the aforementioned wearable sensors. The work by Yakota et al. [281] was not included in the comparison as spin-coating was the primary fabrication method and no printing techniques were used.

### 4.4. Blood Pressure

Regarding major causes of cardiovascular diseases, raised blood pressure or hypertension increases the risk for cardiovascular diseases, is the primary cause of death worldwide, and is the second cause of disability after childhood malnutrition [282]. The WHO estimated that 1.13 billion people have hypertension which is responsible also for increasing the risks of brain, heart and kidney diseases [283]. Blood Pressure (BP) is the pressure exerted by circulating blood on the walls of blood vessels and is considered a vital sign [284]. Hence, high blood pressure or hypertension is defined as the measurement of the maximum blood pressure obtained when the heart contracts or the systolic blood pressure (SBP) is higher than 140 mmHg, and the measurement of the minimum blood pressure obtained when the heart is at rest or the diastolic blood pressure (DBP) is higher than 90 mmHg [282,285]. Barrios et al. [286] summarized the classifications of blood pressure for adults, regarding abnormally low (hypotension), normal, and abnormally high (hypertension) blood pressure.

In this context, blood pressure is a valuable vital sign that needs to be continuously monitored for critically ill and vulnerable patients for clinical management and cardiovascular risk prediction [287]. Blood pressure measurement can be classified mainly into two methods, the non-invasive method and the invasive method. The former requires an arterial line or catheter to insert intravenously into the patient’s artery while the patient is in the hospital. However, this method is highly accurate and provides continuous blood pressure measurements [285,288]. The second can be classified into the auscultatory and oscillometric methods, the Tonometry method, the Volume clamp method and the Pulse wave velocity method. These methods are extensively discussed and evaluated in [289] from the accuracy and performance point of view. Additionally, Tjahjadi et al. [290] classified these methods in map form. Most devices used in clinical settings follow the volume clamp method, but, despite their accuracy, these methods are not comfortable for patients [289].

Continuous and ambulatory beat-to-beat monitoring of blood pressure, due to the high variability of its measurements during the day, in a way that is non-invasive, real-time, accurate, and comfortable, is an attractive research track. With the assistance of ambulatory measurements, obtaining a comprehensive view of the blood pressure condition during daily life could also help to detect true blood pressure in making the right clinical decision [291,292,293]. Therefore, printed pressure sensors are a promising solution for continuous blood pressure measurement due to to their flexibility, portability, and accuracy.

**Figure 7:**
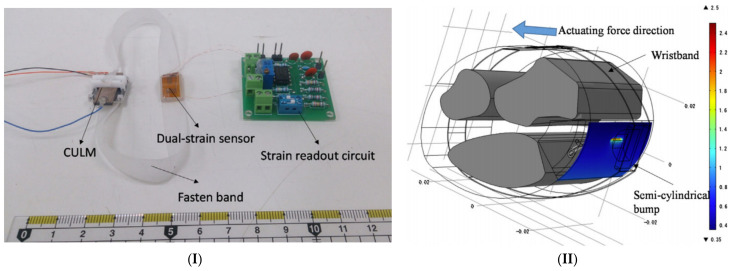
Printed blood pressure monitoring devices from the literature. (**I**) Strain gauges sensor by Wang et al. [294] showing CULM and strain sensor, (**II**) displacement simulation on the skin as a result of the actuation force. Reused with permission, Copyright (2016), Elsevier.

Wang et al. [294] developed a non-invasive, screen printed sensor for continuous estimation of blood pressure measurements based on the concept of diastolic and systolic strain change in wrist skin. A dual strain gauge fabricated on a polyimide substrate and silver paste was printed as the sensing material to detect the low resistance variation resulting from blood pressure. This dual strain sensor was attached to a wristband with a compact ultrasonic linear motor (CULM) to achieve the proper binding force for the indentation depth, as can be seen in Figure 7I, and to obtain the indentation pressure required, based on the wrist elasticity model (Figure 7II). This system was tested on 30 healthy subjects and the results showed that the maximum error at resting on the seat was (−2.0 ± 3.9 mmHg), and this error increased to (−6.9 ± 9.6 mmHg) during exercises, in comparison to a reference cuff-based blood pressure monitor. In contrast, Noh et al. [295] chose a ferroelectric film-based approach to achieve a flexible and non-invasive continuous blood pressure sensor. Their sensor estimated systolic blood pressure from the RJ interval, where R represents the peak of the ECG and J represents the peak of the ballisto-cardiogram (BCG). The proposed sensor consisted mainly of four layers, a bottom layer with screen printed ECG electrodes, an electromechanical film as a common layer, a middle layer that has electrodes to measure the current produced by the film for BCG measurements, and a flexible circuit as the top layer for conditioning the BCG and ECG waveforms. The sensor was tested on three healthy subjects and presented good performance, whereby the estimated SBP agreed with the reference SBP. Table 5 shows a comparison list for the two aforementioned sensors.

Blood pressure is inherently difficult to measure directly in a convenient manner, because a force must usually be applied to the artery. However, due to the current advancement in regression, estimation, and neural network models, blood pressure can be estimated from ECG and PPG signals as has been previously shown in [296,297]. The estimation technique is promising for wearable sensors, as the printed sensors discussed in the heart rate measurement section can be utilized for accurate blood pressure estimation.

## 5. Conclusions

In this review, we pursued and intensive discussion highlighting the integration between the monitoring of common symptoms related to priority infectious diseases and printed wearable sensors as a control weapon, in the era of the epidemic. The top priority infectious diseases which pose an enormous threat to public health with epidemic potential, according to WHO, are COVID-19, CCHF, EBOV and MARV, LHF, MERS and SARS, Nipah Virus disease, and RVF. These diseases were reviewed to highlight the pathogen causing the disease, modes of transmission, case fatality rate, symptoms, diagnosis methods, vaccine availability, and clinical management. On the other hand, a wide literature was reviewed to investigate printed wearable sensors that could be used to monitor vital signs (body temperature, respiratory rate, heart rate, and oxygen saturation) in a non-invasive and comfortable way. Then, the selected sensors were summarized to help researchers in further investigation of the current gaps and to develop high-performance health monitoring devices for effective epidemic disease control. Given enough data, these vital signs records can also be used for other diseases like influenza, asthma and COPD (Chronic Obstructive Pulmonary Disease). Wearable sensors are expected to receive a significant attention due to the urgent need for durable, accurate, and low-priced sensors to be worn daily without triggering discomfort or inconvenience, especially for real-time disease monitoring. Therefore, in addition to the printed wearable sensors discussed, wearable biosensors also provide a huge opportunity and could expand the monitoring from vital signs to analysis glucose level and PH of sweat, tears or saliva, etc. Integrating these sensors into daily life moves from direct attachment to skin towards watch-style bands, textiles or facemasks. However, wearable sensors face common challenges despite important progress. Firstly, extensive successful clinical evaluation studies are needed to obtain the acceptance of the medical community, and this is also related to the lack of data currently available for examining how vital signs development can help in diagnostics or in accumulating a better understanding of disease development. Secondly, there are also challenges in wireless communication, power management and batteries, data security and privacy.

All the proposed diseases in this study were the victims of insufficient research interest before the COVID-19 outbreak occurred; hence, broad research should be carried out to obtain a mature scientific knowledge about infectious diseases, along with wearable sensors to improve the quality of epidemic disease control.

## Figures and Tables

**Table 1:** Summary of reviewed infectious diseases highlighting the pathogen causing the disease, modes of transmission, case fatality rate, symptoms, diagnosis methods, vaccine availability, and clinical management.

Emerging Diseases	COVID-19	CCHF	EBOV, MARV	LHF	MERS, SARS	RVF	NiV
Pathogen	SARS-COV-2 virus [142]	Crimean–Congo hemorrhagic fever virus (CCHFV) [63]	Ebola and Marburg viruses [73]	Lassa virus [68]	MERS-CoV and SARS-CoV for MERS and SARS	Rift Valley Fever Virus (RVFV) [118,119]	Nipah virus (NiV) [130]
CFR	3.4% [143]	10–40% [62]	24–90% [76,78]	1% [86]	MERS 34.4% [112], SARS 11% [113]	Less than 1% [120]	40–75% [131]
Transmission	Respiratory droplets [38,39]	Tick bites, exposure to infected livestock, and human to human transmission [62,65]	Direct contact with contaminated bodily fluids of infected humans and animals [74,75,76]	Direct contact with infected persons or animals and their bodily fluids [86]	Respiratory droplets [94]	Mosquito bites, direct contact with infected animals. [120,121,122]	Direct contact with infected fruit bats, pigs, or other infected animals and their secretions [131]
Symptoms	Fever, cough, shortness of breath, loss of taste and smell [43,45,46,47,48]	Fever, muscle pain, dizziness, vomiting, diarrhea, headache, photophobia [62]	Fever, fatigue, diarrhea, vomiting, hemorrhagic manifestations [80,81]	High fever, general weakness, malaise, sore throat, headache, muscle [88]	Fever, chills, coughing, malaise, myalgia, and headache [93]	Fever, fatigue, and muscle pain [120,122,123,124,125]	Fever, cough, vomiting, sore throat, unconsciousness, and disorientation [131,135,136,137]
Diagnosis	rRT-PCR and antibody detection [49,50,51]	ELISA, serum neutralization, antigen detection and RT-PCR	ELISA, antigen detection, RT-PCR, and virus isolation [76,78]	ELISA, RT-PCR, virus isolation by cell culture and antigen detection [86,89]	rRT-PCR and antibody detection tests [144]	ELISA, virus isolation and RT-PCR. [122,126]	RT-PCR and ELISA [138]
Vaccine	Vaccine is available	No licensed vaccine is available	No vaccines are available [82]	No vaccines are available [86]	No vaccines are available [97,114]	No vaccine is available [120]	No vaccine is. available [130,139,140]
Clinical Monitoring	BT, RR, HR, and SpO_2_. [58,59,60]	BT, HR, BP [62,72]	BT, HR, RR, BP, SpO_2_, consciousness, and glucose [84,85]	BT, BP, HR, and RR, in addition to other laboratory tests [91,92]	BT, SpO_2_, RR, BP, HR, Bodily fluids, and Sepsis [110,115,116,117]	BT and BP are crucial clinical features to be continuously monitored [123,129]	BT, SpO_2_, RR [141]

**Table 2:** Comparison between selected printed temperature sensors.

Group	Position	Printing Method	Substrate	Active Material	Sensing Method	Sensitivity
A. Eshkeiti et al. [230]	Wrist	Screen	PET	Silver	Resistive	N/A
Wang et al. [223]	Arm	Inkjet	PEN	PEDOT:PSS	Resistive	−7.7 m°C^−1^
Ali et al. [224]	Wrist	Inkjet, Screen	Kapton	Carbon	Resistive	3.7 m°C^−1^
Dankoco et al. [225]	N/A	Inkjet	Kapton	Silver	Resistive	2.23 m°C^−1^
Yamamoto et al. [226,228]	Chest	Screen	PET	CNT and PEDOT:PSS	Resistive	13 m°C^−1^
Voutilainen et al. [229]	N/A	Screen	PET	Polymer paste	Capacitive	9 kHz °C^−1^
Han et al. [227]	Wrist	Inkjet	N/A	PEDOT:PSS	Resistive	N/A

**Table 3:** A review of printed respiratory rate sensors.

Group	Position	Printing Method	Substrate	Active Material	Sensing Method	Sensitivity
Al-Halhouli et al. [184,255,256]	Chest, abdomen	Inkjet	PDMS	Silver nanoparticles	Resistive/strain	0.11 S ^−^^1^
Yang et al. [259]	Facemask	Screen	PVDF NM	Silver nanowires	Capacitive/pressure	4.2 kPa^−1^
Cao et al. [260]	Facemask	Screen	PVDF NM	Silver nanoparticles	Triboelectric/pressure	0.065–0.385 kPa^−1^
Lu et al. [262]	Mouth	Inkjet	glossy photo-paper	Graphene	Impedance/temperature, humidity	N/A
Su et al. [261]	Facemask	Inkjet	PET	Gold nanoparticles	Resistive/humidity	N/A
Mohapatra el at. [263]	Nose	Inkjet	Polyimide	Silver nanoparticles	Resistive/moisture	N/A

**Table 4:** A comparison between printed heart rate and SpO_2_ sensors in the literature.

Group	Position	Printing Method	Substrate	Active Material	Sensing Parameter	Error
Yamamoto et al. [226,228]	Chest	Screen	PET	CNT	ECG/3 electrodes	N/A
Vuorinen et al. [279]	Chest	Screen	TPU	Silver	ECG/4 electrodes	N/A
Abu-Khalaf et al. [188]	Finger	Inkjet	PDMS	Silver	HR/SpO_2_	HR: ±4.72%, SpO_2_: ±0.755%
Lochner et al. [280]	Finger	Blade coating, spin coating	PEN, PET, ITO coated glass	Organic materials	HR/SpO_2_	HR: 1%, SpO_2_: 2%

**Table 5:** A comparison of continuous printed blood pressure monitoring devices.

Group	Position	Printing Method	Substrate	Active Material	Sensing Parameter	Error
Wang et al. [294]	Wrist	Screen printing	Polyimide film (PI)	Silver paste	Resistive/strain	−2.0 ± 3.9 mmHg
Noh et al. [295]	Chest	Screen printing	EMFi	Silver paste	ECG/electrodes	−0.16 ± 4.12 mmHg

## Data Availability

Not applicable.

## Supplementary Materials

The following are available online at https://www.mdpi.com/article/10.3390/mi12060620/s1, Excel sheets for the statistics in Figure 1 along with the MATLAB code that plots the figure, figures permissions are also included
